# Pathological changes in the brain after peripheral burns

**DOI:** 10.1093/burnst/tkac061

**Published:** 2023-02-06

**Authors:** Jigang Chen, Danfeng Zhang, Junhui Zhang, Yanni Wang

**Affiliations:** Department of Burn and Plastic Surgery, Beijing Children’s Hospital, Capital Medical University, National Center for Children's Health, Beijing 100045, China; Department of Neurosurgery, Shanghai Changzheng Hospital, Second Military Medical University, Shanghai 200003, China; Institute of Burns Research, State Key Laboratory of Trauma, Burns and Combined Injury, Southwest Hospital, Third Military Medical University, Chongqing 400038, China; Department of Geriatric Oncology, Department of Palliative care, Department of Clinical nutrition, Chongqing University Cancer Hospital, No.181 Hanyu Road, Shapingba District, Chongqing 400030, P.R. China; Department of Burn and Plastic Surgery, Beijing Children’s Hospital, Capital Medical University, National Center for Children's Health, Beijing 100045, China

**Keywords:** Burn, Brain injuries, Pathological changes, Blood–brain barrier, Inflammation

## Abstract

Brain injuries are common complications in patients with thermal burns and are associated with unpleasant outcomes. In clinical settings, it was once believed that brain injuries were not major pathological processes after burn, at least in part due to the unavailability of specific clinical manifestations. Burn-related brain injuries have been studied for more than a century, but the underlying pathophysiology has not been completely clarified. This article reviews the pathological changes in the brain following peripheral burns at the anatomical, histological, cytological, molecular and cognitive levels. Therapeutic indications based on brain injury as well as future directions for research have been summarized and proposed.

Highlights

- Brain injuries are common after burn and have been underestimated.
- Proinflammatory mediators, reactive nitrogen species and reactive oxygen species are associated with burn-related brain injuries.
- Activation of neutrophils and microglia aggravates brain injury after burn.
- Cerebral hemodynamic changes further insult the brain.

## Background

Cerebral disorder has long been recognized as a common problem in patients with thermal burns. ‘Intensive cerebral congestion’ was first described in 1832 in the autopsy of burn victims [1]. This phenomenon was rarely mentioned until 1928, when neurological complications were reported in a child with an extensive second-degree burn [2]. Since then, several hundred cases of pediatric burns with neurological sequelae have been described, with a reported incidence rate ranging from 1% to 85% [3,4]. These patients, with a total body surface area (TBSA) of the burn ranging from 7% to 60%, were indicated to suffer from ‘burn encephalopathy’, which was defined as an acute neurological dysfunction following thermal injury irrelevant to iatrogenic factors [4,5]. Patients with burn encephalopathy usually present with seizures, agitation, hallucination, delirium and other neurologic disturbances. It seems that children are more susceptible than adults to neurological dysfunction after burn, which might be due to the relatively smaller surface area, higher initial metabolic rate and underdeveloped brain [4].

**Figure 1:**
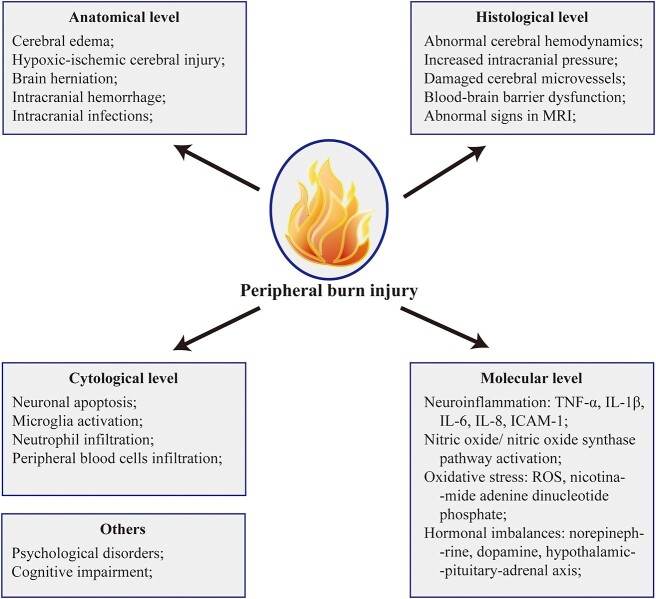
Pathological changes in the brain after peripheral burns. Following peripheral burn injury, different levels of pathological changes could be observed. *MRI* magnetic resonance imaging, *IL* interleukin, *TNF-α* tumor necrosis factor-α, *ICAM-1* intercellular adhesion molecule-1, *ROS* reactive oxygen species

There has been a conspicuous change over the last century in the clinical manifestations, pathological features and outcomes of burn encephalopathy. Earlier reports showed some stormy events, including seizures, obtundation, coma or even respiratory arrest, all of which were apparently related to cerebral herniation [2,6–11]. Autopsy and animal studies have also indicated major pathological changes in the brain, such as severe edema with diffuse parenchymal hemorrhage [12–14]. However, with the improvement of medical and surgical treatment, especially appropriate fluid management, brain edema and herniation were reported to be rare in burn encephalopathy or were not even reported [3,15]. Notwithstanding, a considerable number of burned patients with any severity still showed other neurological disorders, such as acute consciousness disturbance and seizures, at any time from several hours to months after injury. Moreover, in a study involving 5260 patients from a single pediatric burn center, 145 patients died after burn [16]. According to the autopsy reports, brain injury accounted for 16% of all deaths, which ranked only after sepsis and respiratory failure. Of those with brain deaths, anoxic brain injury accounted for 48% and cerebral edema with herniation accounted for 52%. Of note, nearly 1 in 4 patients who died from brain injury had only minor burns. These results suggest that burn-related brain injury remains a critical issue that should not be neglected, especially among children [17].

**Figure 2:**
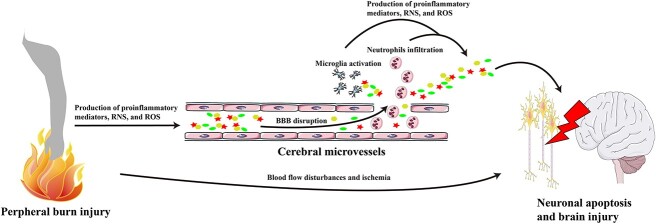
Potential mechanisms of burn-induced brain injury. Following peripheral burn injury, damaged tissues produce large amounts of proinflammatory mediators, RNS and ROS, leading to disruption of the BBB. These molecules, together with neutrophils, further enter the brain by the disrupted BBB and then activate microglia. Proinflammatory neutrophils and microglia increase BBB permeability and facilitate the recruitment of perivascular neutrophils, which in turn aggravate brain injury. Furthermore, blood flow disturbances and ischemia caused by cerebral hemodynamic changes and microvascular damage further insult the brain. *BBB* blood–brain barrier, *RNS* reactive nitrogen species, *ROS* reactive oxygen species

In the past two decades, intensive efforts have been made in the study of brain injury after burn. In the present review, we aim to provide an illustrative summary of pathological changes in the brain after peripheral burns to gain a comprehensive understanding of them (Figure 1 and 2).

## Review

### Pathological changes in the brain after burn injury

#### Anatomical level

A large body of human autopsy data has confirmed the significant pathological changes in the brain after burn. According to gross neuropathological examinations, the major findings were cerebral edema, hypoxic–ischemic cerebral injury, brain herniation, intracranial hemorrhage and infections [6,12,14,16,18,19].

Cerebral edema was the first reported neuropathological finding after burn, which was described by Dupuytren *et al*. in 1832 [1]. Later after that, some early studies indicated that cerebral edema was the uniform and most common finding [3,6,9]. However, no brain weight changes have ever been reported and hypoxic–ischemic cerebral injury might be more common than cerebral edema [19]. In fact, the neurological disturbances that occurred early after burn depended principally upon hypoxia or ischemia [3]. As a result of hypovolemia after burn, cerebral ischemia is a common underlying cause of encephalopathy or death, although it is generally underestimated in examining global indices of circulatory adequacy [20].

Brain herniation is usually the direct cause of death for victims with burn encephalopathy. It is interesting to note that tonsillar herniation, a more fatal type of herniation than others, is the most frequent. For example, Emery and Campbell Reid reported on 7 young children with burn encephalopathy after burns, and 4 of them were found to have herniation or protrusion of the cerebellar tonsils through the foramen magnum at autopsy [6]. Moreover, signs of intracranial hypertension, such as flattening of cerebral convolutions or absence of free cerebrospinal fluid, were indicated in all of them. It appears that minor burns could also lead to brain herniation, as these deceased children only had minor burns with a TBSA ranging from 5% to 11%. In another study, pathomorphological changes in the brain were reported in autopsies of 58 burn patients; 11 of them were indicated to suffer from tonsillar herniation, while only 2 cases were indicated to suffer from temporal herniation [12].

Although the incidence is only one-fifth as frequent as ischemic injury, hemorrhagic complications are still common in burned patients [14]. They are diffusive and generally present as multiple petechial or subarachnoid hemorrhages (SAH) in the brain and spinal cord [12,18]. These hemorrhages sometimes circled the vessels. In the absence of hypoxia and hemodynamic changes, massive microvascular hemorrhaging and bleeding in the brain are more frequent in smoke-inhalation injuries than in skin burns [21]. The consumption of platelets, fibrinogen, plasminogen and clotting factors in the burned tissue or throughout the circulation was considered to be the cause of hemorrhage after burn [14,22,23]. Despite all this, few studies have considered intracranial hemorrhage as a direct cause of death after burn.

Infections are also important complications. Although the incidence of intracranial infection in patients with severe burns is extremely low at 0.1% [24], it has been reported that 15.8% of patients who die from burn injuries have intracranial infections [14]. Candida species (microabscesses in the brain), *Pseudomonas aeruginosa* (meningitis) and *Staphylococcus aureus* were the three dominant responsible microorganisms. Cerebral infections usually develop in the second or third weeks after injury. They were considered to be complications of systemic infections derived from burn wound infection, endocarditis and pneumonia, since all these patients tested positive in their blood germicultures [14].

#### Histological level

##### Cerebral hemodynamics and intracranial pressure

Hemodynamic change is a hallmark of burn patients, especially for those with severe burns. It can be divided into two phases: the resuscitation phase and the hyperdynamic and hypermetabolic phase [25]. The resuscitation phase, usually known as the ‘ebb phase’ or ‘hypodynamic phase’, occurs immediately after the injury and typically lasts for 1 to 3 days [25,26]. After injury, there is a significant loss of circulating plasma volume due to increased capillary permeability, which is derived from vascular injury and the release of inflammatory mediators [27]. As a result, edema emerges in both burned and unburned tissues, followed by the depletion of intravascular volume, reduced cardiac output and increments in systemic vascular resistance [25,28,29]. After burn, systemic vascular resistance increased two to three times compensatorily to preserve heart rate and systolic blood pressure [30]. Therefore, the primary goal of therapy during this phase involves maintaining tissue perfusion with adequate fluid resuscitation. Subsequently, the hyperdynamic and hypermetabolic phase, also known as the flow phase, starts nearly 3 to 5 days after injury [26,31,32]. This phase is characterized by decreased capillary permeability, decreased peripheral vascular resistance, and increased heart rate and cardiac output [25,31,33]. During this phase, patients require much less fluid input compared to the previous phase.

Cerebral circulation is an integral part of systemic circulation. However, the hemodynamic changes in cerebral circulation are significantly different from those in systemic circulation [20,34]. Such a difference is largely due to the autoregulation of cerebral blood flow, which is able to remain relatively constant despite changes in systemic perfusion pressure [34]. An ovine model of severe scald injury (70% TBSA) proved that while cardiac output decreased immediately after burn, the blood flow of the whole brain increased [20]. Then, even with adequate fluid resuscitation, the cerebral blood flow decreased by 6 hours after injury, which might be the result of the damaged blood flow autoregulatory ability of the brain. Unlike cerebral blood flow, cerebral vascular resistance decreased at first and then increased above the baseline value by 6 hours. Burns have a significant impact on the level of intracranial pressure, which increases continuously after injury [20,35]. The elevated intracranial pressure was more likely to be the result of encephaledema rather than arterial carbon dioxide tension, as it remained constant postburn [20].

##### Cerebral microvessels

Increased microvascular permeability is one of the main characteristics of burn injury and it has been clearly observed in the peripheral vessels [36]. Several studies have also investigated the cerebral vascular response to systemic thermal injury. Moati *et al*. isolated a lipoprotein fraction presented in Cohn fractions II and III from the sera of patients with at least 30% TBSA covered by third-degree burns [37]. After intraventricular injection into rats, the fraction induced increased blood–brain barrier (BBB) permeability, which suggested that toxic substances from the serum of burned patients could lead to increased permeability of the cerebral microcirculation. With *in vivo* visualization of cerebral microcirculation by injection of fluorochrome fluorescence isothiocyanate (FITC) albumin into the vein of rats with a 70% TBSA third-degree burn, Barone *et al*. found significant albumin leakage in the cerebral vessels, progressive arterial dilation and an increase in permeability from 20% at 15 min to 104% at 6 h [38,39]. These experiments demonstrated for the first time the changes in cerebral permeability after severe systemic thermal injury. Hu *et al*. observed the opening of the BBB from 2 h after injury in a model of 50% TBSA third-degree scalded rabbits with transmission electron microscopy [40]. This result suggested that BBB opening during the early stage of burn injury might be a prerequisite for cerebral edema. BBB damage was accompanied by morphological changes in cerebral microvessels. Significant progressive arterial dilatation over 6 h was demonstrated in thermally injured animals [41,42]. Moreover, the distributional density of capillaries, as well as their volume and surface fractions, increased at 6 and 12 h after burn injury, while they decreased at 18 h and reached the lowest point at 24 h [43,44].

##### B‌BB dysfunction

The underlying mechanisms of BBB damage after burn injury have been explored. After exposure to burn injury, a plethora of proinflammatory mediators, such as interleukin 1-β (IL-β), IL-6, IL-8, tumor necrosis factor-α (TNF-α) and intracellular cell adhesion molecules, increase dramatically in both peripheral and cerebral circulation [45–48]. BBB collapse after peripheral burn injury is caused by the upregulation of proinflammatory mediators [17,47]. Numerous studies on cerebrovascular and neurological disorders have confirmed damage to the BBB in a cytokine/chemokine-dependent manner [49,50]. The activation of several other molecules, such as matrix metalloproteinase (MMP), tissue plasminogen activator, urokinase plasminogen activator, reactive nitrogen species (RNS) and reactive oxygen species (ROS), has also been suggested to play a key role in BBB disruption after different degrees of burns [17,51–54].

Molecular changes other than proinflammatory cytokines, RNS and ROS after peripheral thermal injury induce BBB dysfunction. The basement membrane components of the BBB are degraded by a family of proteases known as MMPs. They are produced by various cells, such as endothelial cells, microglia and astrocytes, in response to injury [55]. Upregulation of MMP-2 and MMP-9 mRNA in brain tissues was reported as early as 3 h following a third-degree scald injury affecting 60–70% of rat BSA [51,52,54]. The results suggested that these proteases might play a key role in BBB disruption.

Aquaporin-4 is the main water channel protein expressed in the brain and is important in regulating cerebral water and potassium homeostasis. Changes in aquaporin-4 have been implicated in several cerebral disorders, including edema, stroke, glioma and traumatic brain injury (TBI) [56]. Results from rats with a 30% TBSA third-degree scald injury showed that cerebral water content and expression of aquaporin-4 increased at 2 h, peaked at 6 h and was still at a higher than normal level at 48 h post-burn [57]. This study indicated that aquaporin-4 promoted an influx of water across the BBB and the formation of brain edema after severe burn.

##### Pathological changes present on magnetic resonance imaging

Magnetic resonance imaging (MRI) has been widely used to evaluate brain injury, with obvious advantages in submillimeter morphological images and high resolution. MRI is more sensitive than computed tomography in examining axonal injury, small hemorrhages and ischemia [58,59]. Furthermore, with the emergence of advanced imaging, such as perfusion-weighted imaging (sensitive to abnormal blood supply and perfusion), diffusion-weighted imaging (sensitive to edema) and functional MRI (fMRI, sensitive to changes in blood flow and oxygen levels), MRI can evaluate a range of morphological and functional targets as an information-rich tool for studying neuropathological changes.

Signs of edema are the most prominent findings on brain MRI after burn. In canine models of 50% TBSA third-degree burns, a 1.5 T brain MRI scan detected diffuse cerebral parenchyma swelling, morphological changes and disappearance of the partial boundary between the gray and white matter at 24 h post-burn [43]. In another study with a 50% TBSA third-degree scalded model of rabbits, the apparent diffusion coefficient values, which were calculated from diffusion-weighted imaging, decreased significantly in the temporal cortex, posterior cortex and basal ganglia at 4 h postinjury. The decrease in the apparent diffusion coefficient values suggested swelling of a large number of cerebral cells in an aggressive manner [40].

Blood oxygen level-dependent fMRI (BOLD-fMRI) has been applied to evaluate postburn brain injury. It depicts changes in deoxyhemoglobin concentration consequent to spontaneous or task-induced modulation of neural metabolism. BOLD-fMRI relies on regional differences in cerebral blood flow to detect the activated area of the brain, as blood flow is highly related to oxygen and carbon dioxide tension in brain tissue [60,61]. In an animal study, BOLD-fMRI was performed for rabbits with 50% TBSA third-degree scaled injury, and changes in whole brain Kendall’s coefficient (ReHo value) and water content were calculated. Significant changes in ReHo values were found in different areas of the brain and were positively associated with brain water content. These results indicated that the neurological impairment after severe burns might be induced by cytotoxic brain edema [62] (in Chinese). In a study involving patients with brain injury after different degrees of burns, BOLD-fMRI detected abnormal signals in the brains of all patients, demonstrating its potential value in evaluating burn-induced brain injury [63]. Furthermore, for patients with posttraumatic stress disorder after severe burn, BOLD-fMRI showed decreased connectivity of the default mode network in the area of the dorsal medial prefrontal cortex, precuneus/posterior cingulate cortex and posterior part of the right superior temporal sulcus [64]. This decreased connectivity might be attributed to psychological dysfunction after injury.

#### Cytological level

##### Neuronal apoptosis

Peripheral burn injury can lead to neuronal apoptosis. In burn-injured animal models, neurons underwent this orchestrated form of cell death characterized by the swelling of cell bodies, axons and mitochondria, as well as a decrease or disappearance of Nissl bodies, concentration of axoplasm, and expansion or vacuolation of the Golgi complex [42,65,66]. Sometimes, neurons are shrunken with condensed nuclei during the early stage of apoptosis [65]. Neuronal apoptosis was also observed in the spinal cord remote from the burn site. For example, Wu *et al*. found that local third-degree hindpaw burn induced apoptosis of spinal cord ventral horn motor neurons and consequently caused skeletal muscle wasting and denervation atrophy [67]. Similarly, in another study, investigators demonstrated that a 35% TBSA third-degree burn injury of the body produced distant effects on the spinal cord and led to ventral horn motor neuron apoptosis and degeneration [68].

##### Microglial activation

Microglia are activated in response to various pathological changes, such as injury, ischemia and infection [69]. As a major source of neuroinflammation, activation of microglia after burn injury has been reported. After staining intracranial microglia in the 15% TBSA full-thickness scald-injured mice with the microglial marker Iba-1, a kinetic change in Iba-1-immunoreactive cells was observed in the cortex and striatum, which were well ramified in burned mice compared to controls. Microglial activation is considered to be associated with delayed neuronal apoptosis [65]. Similar activation of microglia in the brain and spinal cord, together with their potential role in neuroinflammation and neuronal apoptosis, has also been suggested in several other studies [65,68,70].

##### Neutrophil infiltration

Neutrophils are activated after severe burn injuries [36]. Damaged BBB and increased microvascular permeability allow systemic neutrophils to infiltrate freely into the perivascular space. This process is usually driven by some intracerebrally produced cytokines, such as TNF-α, IL-1β and IL-6 [32,71]. Adhesion molecules such as intercellular adhesion molecule-1, vascular cell adhesion molecule-1 and E-selectin are needed for neutrophil infiltration to facilitate endothelial adherence and transit into the brain parenchyma [72,73]. These adhesion molecules increased significantly after severe burn injuries [74,75]. Neutrophil infiltration into the brain occurs as early as 8 h postburn, followed by progressive infiltration, which usually leads to dispersed infiltration or even microabscesses [65,70,76]. These activated and infiltrated neutrophils further release proteases, RNS, ROS and cytokines, aggravating the neuroinflammatory response [77,78].

#### Molecular level

##### Inflammation

Severe burn injuries result in the rapid production of proinflammatory cytokines by damaged tissues, leading to increased serum levels of different cytokines, such as TNF-α, IL-1β and IL-6 [45,79]. These locally produced cytokines then become systemic and orchestrate the inflammatory cascade in response to injury by regulating the expression of adhesion molecules responsible for neutrophil infiltration [80], attacking the BBB and impairing vascular permeability. In addition, IL-6 is indicated to contribute to intracranial hypertension [81,82]. Pro-inflammatory cytokines and adhesion molecules could also be produced intracerebrally, as there is significant upregulation of mRNA levels of TNF-α, IL-1β and intercellular adhesion molecule-1 in brain tissue at 3 h following a third-degree thermal injury affecting 60–70% of the BSA [47]. Although the exact source of cytokines released in the brain is unknown, infiltrated neutrophils and activated microglia have been implicated [69,83]. Inflammasomes are another source of proinflammatory cytokines [84], but their role in neuroinflammation after burn injury remains to be elucidated.

##### Nitric oxide/nitric oxide synthase pathway

Nitric oxide (NO) is a major physiological messenger regulating vasodilation, immunity, neurotransmission and various pathophysiological situations [85]. This gaseous compound is biosynthesized from L-arginine by different isoforms of NO-synthase (NOS) enzymes localized in endothelial cells, neurons and mitochondria [86]. Pathological alterations in the NO and NOS pathways occur early after burn injury. The urinary level of the stable NO metabolite is elevated for >1 week after a severe scald injury, which could be inhibited by an NOS inhibitor [87]. Furthermore, increased levels of NOS activity are also detected in the liver, kidney, spleen and gastrointestinal tract following scald insult, and the injured skin has greater NOS activity [88].

In contrast to other organs and tissues, however, the cortical level of NO and mRNA expression of inducible NOS in the hypothalamus decline rapidly following severe burns [89,90]. The reason for this discrepancy remains to be further explored. A similar decrease in cerebral NO levels is also observed in animal models of SAH [91]. This suggests that the decrease in cerebral NO levels involves scavenging by hemoglobin, free radicals and vascular nitrite reduction rather than impairment of NO synthesis [92].

##### Oxidative stress

Under normal conditions, free ROS derived from radicals exist in biological cells and tissues at a low concentration, and there is an exquisite balance between the production and destruction of ROS [93,94]. Inflammatory cells such as neutrophils and microglia can produce ROS [95]. Many studies have demonstrated that burns induce systematic inflammatory reactions by producing ROS and ultimately lead to peroxidation [96–98]. ROS generated after burn injury include superoxide anions, hydroxyl radicals, hydrogen peroxide and peroxynitrate [77]. Among numerous sources of ROS, neutrophil nicotinamide adenine dinucleotide phosphate oxidase constitutes the major cellular source of ROS in burn-injured tissues [77]. Neutrophil infiltration and accumulation in the remote tissues of burn injury are involved in the pathogenesis of remote organ damage by the production of ROS [99]. Among different organs, the brain is particularly susceptible to oxidative stress [100]. Animal studies demonstrated that after severe burn injuries, there was a significantly elevated level of the end product of lipid peroxidation, malondialdehyde, and decreased levels of the antioxidant superoxide dismutase as well as mitochondrial glutathione [101,102]. The consequences of oxidative stress include injury to the endothelium of the vascular wall, damage to the BBB and activation of the apoptotic pathway [103–105].

##### Hormonal imbalances

Severe thermal injury is followed by a pronounced hypermetabolic response that persists for up to 2 years [106,107]. The hypermetabolic response is characterized by increased metabolic rates, insulin resistance, multiorgan dysfunction and increased risk of infection [106–109]. This response is, at least in part, mediated by up to 50-fold elevated levels of plasma catecholamines and cortisol [110]. In addition, there are increased levels of norepinephrine and dopamine in certain areas of the brain after severe burn [111–113]. Blockade of β-adrenergic receptors with propranolol has been demonstrated to attenuate hypermetabolism and reverse muscle-protein catabolism among severely burn-injured children [114,115]. Alterations in hypothalamic function and disturbance of the hypothalamic–pituitary–adrenal axis in severely burned patients have also been described in several studies [116–118].

#### Psychological disorders and cognitive level

Psychological distress is among the most prevalent and crippling complications of major burn injuries with long-term consequences. Reports based on the Burn Model Systems dataset indicated that in-hospital psychological distress occurred in 34% of patients with major burns, and one-third of them had significant psychological distress at discharge [119,120]. Clinically significant symptoms of stress disorders and depression are common. The incidence of acute stress disorder was reported to be 18–26% across different countries [121–123]. Approximately one-third of victims of major burns were diagnosed with posttraumatic stress disorder in Japan and the USA at 3–6 months postinjury [122,124]. Several studies have reported that nearly 20–40% of burn patients experience different degrees of depression [121,125–127]. In addition, sleep disturbances such as nightmares and insomnia have been frequently reported as long-term sequelae after burn [128–130].

The event that leads to burn injury is often traumatizing, and rescue, hospitalization and treatment can be scary experiences for children [131]. Hospitalization and treatment often involve separation from family and parents, which is itself traumatic [132]. Furthermore, burn injuries, as well as subsequent wound care, are both physically and mentally painful. This is especially true for children who may not understand the necessity of procedural pain as a part of recovery [133]. The most commonly reported psychological disorder of pediatric burn patients is anxiety, followed by traumatic stress, depression/mood disturbances and emotional issues [134]. These findings highlight the fact that pediatric patients are particularly vulnerable to the psychological impact of burns [134].

Although the literature frequently emphasizes mental consequences and corresponding therapies, cognitive impairment as a potential comorbidity in the burn-injured population is often overlooked and underestimated. Severe burn injuries are usually associated with significant cognitive deficits [135,136]. As described above, there was a significantly increased level of neuroinflammation following burn insult. Inflammatory mediators play a critical role in hippocampal neurogenesis and memory formation [137], and both observational epidemiological studies and clinical trials have demonstrated significant associations between neuroinflammatory markers and cognitive impairment [138]. An animal study demonstrated that cerebral NO changes after burn injury are likely to account for cognitive impairment in rats [90]. Additionally, anoxia, toxic fume inhalation, dehydration and hypoperfusion are among other potential factors contributing to cognitive dysfunction.

### Therapeutic indications based on brain injury

#### Overall treatment targeting burns

Treatment of burns involves first aid, burn size and depth assessment, fluid resuscitation, wound excision, infection control, grafting and coverage, and nutrition support. The mortality of severely and extensively burned patients has decreased greatly over the last 80 years due to progress in treatment based on research findings of burn pathophysiology [139,140]. In particular, early fluid resuscitation and wound excision play an important role in reducing the morbidity and mortality of burn victims by maintaining circulatory volume and cardiac output, ensuring adequate oxygen supply, alleviating the inflammatory response and accelerating wound healing, all of which contribute to the prevention or recovery of burn-related brain injuries [141,142].

It is understandable that the undoubted benefits of early fluid resuscitation and wound excision are closely related to the aforementioned neuropathological changes after burn. Significant loss of fluid occurs immediately after the major burn and frequently leads to hypodynamia and hypovolemia. The primary goal of early fluid resuscitation is to restore circulating volume and cardiac output, improve tissue and organ perfusion, and finally ensure blood supply. Even though cerebral circulation is different from systemic circulation in hemodynamics after burn, normal systemic circulation is indispensable for the brain to restore normal blood supply. In addition, as mentioned above, the systemic inflammatory response triggered by local injury results in a vicious cycle of pathological changes. Disruption of the downward systemic inflammatory response by early aggressive debridement may thus help restore neurological function and reduce morbidity and mortality.

#### Treatment targeting secondary brain injury

Currently, there is a paucity of literature investigating the treatment of burn-induced brain injury. However, as we have indicated above, burn-induced brain injury is a secondary brain injury. It shares similar pathological processes, such as cerebral edema, disruptions to the BBB, neuroinflammation, neuronal apoptosis and oxidative stress, with secondary brain injury triggered by other insults, including TBI, intracerebral hemorrhage or SAH [92,143,144]. Therefore, experience might be borrowed from the treatment of secondary brain injury induced by other insults, especially the treatment targeting specific pathological mechanisms.

Numerous studies have been conducted to identify an effective way to combat brain edema, neuroinflammation, neuronal apoptosis, oxidative stress, etc. [145,146]. For example, in rats with TBI, erythropoietin (EPO) therapy suppresses neuroinflammation with a significant downregulation of proinflammatory cytokines [147]. EPO also showed anti-apoptotic effects by upregulating the phospho-Akt protein. However, clinical trials involving moderate to severe TBI showed that EPO did not improve the functional outcomes of these patients [148]. Most preclinical experiments and phase I/II clinical trials have shown positive effects, but almost all of them have failed in phase III clinical trials. More than 30 clinical studies of pharmaceutical agents for the treatment of TBI have failed over the last three decades [143]. Such failure to translate successful animal experiments to clinical settings is common in brain injury induced by trauma, stroke and hemorrhage [92,149,150]. Several factors, such as different animal species and methodological flaws in animal studies, might have made the translation of animal research results into clinics difficult. Perhaps time will tell regarding the usefulness of animal models for informing clinical trials.

#### Animal studies on the treatment of burn-induced brain injury

A few animal studies have focused on the treatment of burn-induced brain injury by targeting different molecular pathways. For example, Gatson *et al*. showed that subcutaneous injection of estrogen significantly decreased the levels of TNF-α, IL-1β and IL-6 in the brain tissues of thermally injured rats [71]. In addition, estrogen increased the levels of phospho-extracellular signal-related kinase (ERK) and Akt in brain tissue and blocked the activation of caspase-3 and subsequent poly adenosine diphosphate-ribose polymerase (PARP) cleavage [71]. Thus, estrogens suppress neuroinflammation and apoptosis following severe burn injury and may provide a novel clinical strategy for burn-induced brain injury. Other promising molecular targets, such as sphingosine 1-phosphate, pentoxifylline, gelsolin and captopril, have been reported in different animal studies to maintain BBB integrity, suppress inflammation, reduce apoptotic signaling and alter neuronal nitricoxide synthase (nNOS) expression [65,66,151–153].

### Future directions

A large body of evidence suggests the importance of burn-related injuries in the brain, which arise from a multitude of potential factors. However, the evidence is largely from preliminary research, and several puzzles need to be unraveled in future studies. First, the correlation between burn severity and injuries in the brain is not understood. Mild burns can also lead to significant brain injuries, which has been indicated in some human studies [6,12,16]. Perhaps some key factors arising from peripheral burns that can lead to brain injuries have not been elucidated. Second, the role of some innate cells of the brain in the complex secondary injury cascade needs to be further investigated. For example, microglia have been indicated to be the major contributors to secondary brain injury after TBI, intracerebral hemorrhage and SAH, and they are suggested to be potential therapeutic targets [154–156]. While microglia are activated following burn injury, there is a lack of information about their role in the development of brain injury and whether they can be targeted for prevention and treatment. Third, as we have mentioned above, cognitive impairment after burn is usually overlooked and it often has long-term unpleasant effects on burn victims. While cognitive impairment arises from multiple factors, its direct relationship with brain injury following burn needs to be established. Fourth, children seem to be more susceptible than adults to neurological disorders after burn and more focus should be given to pediatric victims with burn-induced brain injury in future clinical trials.

## Conclusions

Brain injury is common after peripheral burn injury and the underlying mechanisms are complex. Proinflammatory mediators, RNS and ROS released from thermally injured tissues can attack and damage the BBB. These molecules, together with neutrophils, further enter the brain by the disrupted BBB, causing microglial activation and neuronal apoptosis. Moreover, cerebral hemodynamic changes and microvascular damage induce blood flow disturbances and ischemia, which further insult the brain. Other factors, such as infection and hormone imbalance, also contribute to brain injury. We propose that knowledge of cerebral changes after burn injury is crucial for developing effective strategies to mitigate injury.

## Abbreviations

BBB: Blood–brain barrier; BOLD-fMRI: blood oxygen level-dependent functional magnetic resonance imaging; EPO: erythropoietin; IL-β: interleukin β; MMP: matrix metalloproteinase; MRI: magnetic resonance imaging; NO: nitric oxide; NOS: nitric oxide synthase; RNS: reactive nitrogen species; ROS: reactive oxygen species; SAH: subarachnoid hemorrhage; TBI: traumatic brain injury; TBSA: total body surface area; *TNF-α*: tumor necrosis factor-α.

## Funding

Natural Science Foundation of China (82101433).

## Authors’ contributions

JC designed the study and prepared the manuscript. DZ and JZ searched the literature. YW reviewed the manuscript.

## Consent for publication

All authors agreed to the submission and publication of the study.

## Competing interests

None declared.
